# Mulberry leaf extract improves glycaemic response and insulaemic response to sucrose in healthy subjects: results of a randomized, double blind, placebo-controlled study

**DOI:** 10.1186/s12986-021-00571-2

**Published:** 2021-04-15

**Authors:** Pariyarath Sangeetha Thondre, Helen Lightowler, Lis Ahlstrom, Andrew Gallagher

**Affiliations:** 1 Oxford Brookes Centre for Nutrition and Health, Faculty of Health and Life Sciences, Oxford Brookes University, Gipsy Lane Campus, Headington, Oxford, OX3 0BP UK; 2 Phynova Group Ltd, 16 Fenlock Court, Blenheim Office Park, Long Hanborough, OX29 8LN UK

**Keywords:** Mulberry leaf extract, Glycaemic response, Insulinaemic response, 1-deoxynojirimycin, Lifestyle changes

## Abstract

**Background:**

There are many benefits of maintaining healthy blood glucose levels, and studies have shown that lifestyle changes such as changes to diet can successfully restore normoglycaemia in participants with dysglycaemia. Significant health-related lifestyle changes are often difficult to implement and functional ingredients that can reduce glycaemic and insulaemic responses may help at risk populations. The aim of this study was to investigate whether a mulberry leaf extract could lower the glycaemic and insulinaemic responses to 75 g sucrose in healthy individuals.

**Methods:**

A double-blind, randomised, placebo-controlled, crossover design trial was conducted by the Oxford Brookes Centre for Nutrition and Health. Thirty-eight participants were recruited into the trial and, after an overnight fast, were given 75 g sucrose + white mulberry leaf extract, or 75 g sucrose alone. Capillary blood samples were collected at 15-min intervals in the first hour and at 30-min intervals over the second hour to determine glucose and plasma insulin levels. Data analysis was conducted using a paired samples T test or a Wilcoxon signed rank test.

**Results:**

The addition of mulberry leaf extract to sucrose resulted in a significantly lower glycaemic response and insulinaemic response compared to a matched placebo (sucrose alone). The change in blood glucose measurements were significantly lower at 15 min (p < 0.001), 30 min (p < 0.001), 45 min (p = 0.008), and 120 min (p < 0.001) and plasma insulin measurements were significantly lower at 15 min (p < 0.001), 30 min (p < 0.001), 45 min (p < 0.001), 60 min (p = 0.001) and 120 min (p < 0.001). The glucose iAUC (− 42%, p = 0.001), insulin iAUC (− 40%, p < 0.001), peak glucose (− 40.0%, p < 0.001) and peak insulin (− 41%, p < 0.001) from baseline were significantly lower for white mulberry leaf extract compared with the placebo*.* White mulberry leaf extract was well tolerated and there were no reported adverse events.

**Conclusions:**

Mulberry leaf extract can be used as part of lifestyle changes that may lead to healthy blood glucose levels.

*Trial registration*: ISRCTN99601810 (23 October 2020, retrospectively registered)

## Background

Non-communicable diseases (NCDs) account for more than 70% of global deaths, with cardiovascular disease, cancer, chronic respiratory diseases and type-2 diabetes being the most prevalent conditions [1]. NCDs are often preventable through behavioural changes that reduce risk factors such as low levels of physical exercise, consumption of an unhealthy diet and the use harmful substances such as alcohol and tobacco [2]. Controlling and preventing NCDs is of increasing importance as the interaction between coronavirus disease 2019 (COVID-19) and NCDs may exacerbate the burden of disease on healthcare systems. COVID-19 is associated with cardiovascular diseases and with ageing populations around the world, with many individuals who may have multiple chronic co-morbidities, there exists the scenario where there are exponential increases in hospitalizations due to complications between COVID-19 and pre-existing NCDs [3].

One of the predominant NCDs is type-2 diabetes mellitus (T2DM). Globally, the number of people with diabetes mellitus has quadrupled in the past three decades, with more than 90% of those cases being T2DM [4]. The International Diabetes Federation estimates that there are 463 million adults with diabetes [5] with estimates that an additional 7.3% of the world’s adult population being considered as prediabetic with either impaired fasting glucose (IFG) or impaired glucose tolerance (IGT) [6]. Although there are elements of genetic predisposition to T2DM, an unhealthy diet and sedentary lifestyle are contributing drivers of the disease [7]. Many cases of T2DM could however be prevented with lifestyle changes [4]. The Diabetes Prevention Program (DPP) demonstrated that lifestyle modification led to a 58% reduction in diabetes in at risk groups that had IGT [8] and the ten-year follow-up of the DPP study showed that the prevention or delay of T2DM can persist for at least ten years with lifestyle intervention [9].

Dietary supplements are often used to support lifestyle changes. *Morus alba* L. (common name white mulberry) is a small deciduous tree belonging to the Moreae tribe of the Moraceae (common name mulberry) family of flowering plants and extracts of *M. alba* have a long history of use as traditional medicines [10–12]. *M. alba* leaves are rich in carbohydrates and protein, as well as many vitamins and minerals such as beta-carotene, iron, calcium, and zinc. They also possess various polyhydroxy alkaloids, stilbenoids (such as resveratrol and oxyresveratrol), flavonoids (including quercetin and kaempferol), and anthocyanins [13, 14]. The polyhydroxylated alkaloids found in *M. alba*. belong to the chemical class called iminosugars or azasugars and are one of the characteristic identifying compounds found in Morus spp. The most predominate iminosugar in *M. alba* is the piperidine iminosugar 1-deoxynojirimycin (DNJ), a D-glucose analogue with a nitrogen group replacing the oxygen on the pyranose ring [15]. Due to their structural similarity with carbohydrate monosaccharides, iminosugars such as DNJ are able to competitively inhibit carbohydrate-digesting enzymes such as α-glycosidase and amylase [16].

There are a number of published studies showing the beneficial effects of *M. alba* on blood glucose and a recent meta-analysis found that *M. alba* leaf extracts significantly reduced postprandial glucose (PPG) and that its use was safe and well tolerated [17]. Reducose^®^ is a proprietary food ingredient, made from a water extract of *M. alba* leaves, and can be use in dietary supplements or directly blended into functional foods and drinks. A previous randomised, placebo-controlled dose-ranging study showed that it significantly lowers the PPG and postprandial plasma insulin following a starch challenge [18]. As different carbohydrates have different glycosidic bonds linking their constituent monomers together, with different digestive enzymes responsible for their hydrolysis, the aim of this study was to conduct a placebo controlled clinical trial using sucrose (α-1,2 glycosidic bond) rather than starch (α-1,4 glycosidic bond), which was used previously. The previous study tested the benefits of Reducose mulberry leaf extract in dietary supplement form, administered in capsules. Reducose can also be used as an ingredient for functional foods and beverages, and so this study explored the effect of Reducose^®^ when incorporated directly into a beverage containing a high amount of sugar. The previous study showed that the bioavailability of Reducose is important for efficacy, an observation this study sought to confirm by evaluating the effects of Reducose when it is immediately bioavailable in a liquid solution, rather than being taken in the form of a capsule or tablet, which has to dissolve to become bioavailable.

In light of the above, the primary objective of this study was to determine the effect of a single dose of Reducose^®^ mulberry-extract (Reducose^®^) versus placebo on blood glucose and insulin responses when co-administered in a water solution containing 75 g sucrose in normoglycaemic adults. The secondary aim was to show that Reducose^®^ when co-administered with oral sucrose will not disproportionately increase the incremental area under the curve for plasma insulin concentration over 120 minutes in normoglycaemic adults compared to co-administration with placebo.

## Research design and methods

### Study design and setting

A double-blind, randomised, repeat measure, crossover design trial was used to study the glycaemic response (GR) and insulinaemic response (IR) to a proprietary mulberry leaf extract (Reducose^®^) compared with a placebo. Participants acted as their own controls. The trial was conducted by the Oxford Brookes Centre for Nutrition and Health at Oxford Brookes University. Ethical approval for the study was obtained from the University Research Ethics Committee (UREC) at Oxford Brookes University (UREC Registration No: 140806 for glycaemic response and UREC Registration No: 110594 for insulinaemic response). Participants were given full details of the study protocol and the opportunity to ask questions before giving written informed consent prior to participation.

### Participants

Thirty-eight healthy men and women participated in the study (Table 1) and were recruited from Oxford Brookes University, Oxford, UK and from the local community between April 2016 and August 2016.

**Table 1:** Physical characteristics of study population with complete GR and IR data (n = 36)

Variable	Mean ± SD
Age (year)	31.8 ± 10.6
Height (m)	1.69 ± 0.08
Weight (kg)	66.9 ± 10.1
BMI (kg/m^2^)	23.3 ± 2.3
Fat mass (%)	23.5 ± 8.9
Lean body mass (kg)	51.3 ± 9.9
Waist circumference (cm)	80.6 ± 7.3
Hip circumference (cm)	100.2 ± 6.1

Values are mean ± SD

*BMI* body mass index

Participants were included in the study if they were: [1] aged between 18–60 years, [2] had a BMI between 20 and 29.9 kg/m^2^, [3] had fasting blood glucose < 6.1 mmol/l. Participants were excluded from the study if they: [1] were women who were pregnant or lactating; [2] had any known food allergies or intolerance to mulberry leaf extracts; [3] had a pre-existing medical condition or taking medication known to affect glucose regulation and/or influence digestion and absorption of nutrients; [4] had a history of diabetes mellitus (type I/II) or they used antihyperglycaemic drugs or insulin to treat diabetes or related conditions; [5] used steroids, protease inhibitors or antipsychotics medicines as these drugs are known to impact glucose metabolism and body fat distribution.

For all participants, anthropometric measurements were made in the fasted state during the first session. Height was recorded to the nearest centimetre using a stadiometer (Seca Ltd, UK), with participants standing erect and without shoes. Body weight was recorded to the nearest 0.1 kg, with participants wearing light clothing and no shoes. Body fat percentage was measured using a body composition analyser (Tanita BC-418 MA; Tanita UK Ltd).

### Randomisation

Researchers recruiting the participants were unaware of the allocation sequence (concealed allocation). Participants were assigned to a participant number according to their chronological order of enrolment in the study. The allocated participant number was used to identify the participants and their corresponding intervention sequence.

The reference product and test products were administered to participants in a randomised, repeated measures design. In each participant, the reference product and test products were tested on separate days, with at least a two-day gap between measurements to minimise carry over effects.

### Test product

Reducose^®^ mulberry leaf extract (batch number IM150525) was manufactured for the Sponsor (Phynova Group Ltd, Long Hanborough, UK) by Purapharm Pharmaceuticals Co. Ltd (Nanning, China). Reducose^®^ is a commercially available aqueous extract of the leaves of *Morus alba* Linn., standardised to contain 4.5– 5.5% DNJ, as determined by high performance liquid chromatography. The raw mulberry leaf material was analysed for DNJ content, heavy metals, pesticide residues, yeast and molds and was then air-dried by the raw material supplier. The dried mulberry leaf then underwent aqueous extraction and ion exchange chromatography to enrich the alkaloid components. The eluent was reduced under vacuum and passed through ultrafiltration filters prior to spray drying. Maltodextrin was added as an excipient to aid spray drying and to standardise the extract to contain 5% DNJ.

The test and placebo products were manufactured by Chrysalis Health and Beauty Ltd (Nottingham, England). The test and placebo products were placed into 500 mL brown bottles and sealed with screw down caps with tamper-evident seals. The test product contained 75 g sucrose co-blended with 250 mg Reducose^®^ mulberry leaf extract. The placebo product contained 75 g sucrose alone. On the day of testing, 250 mL water was added to the 500 mL brown bottles and mixed through vigorous shaking. The Sponsor provided the treatments and bottled water. Both treatments were the same in terms of taste, appearance and odour. All products were stored in the test kitchen at the Oxford Brookes Centre for Nutrition and Health where the temperature was monitored and recorded daily.

The study products were blinded to both investigator and participants.

### Sample size

A previous study [18] in 37 healthy individuals using 250 mg Reducose^®^ or matched placebo showed a difference in mean incremental area under the curve (iAUC) of 43.1 mmol/l/min between treatment groups. Based on a standard deviation of 53.02 mmol/l/min, a sample size of 34 participants would provide 90% power to detect a similar treatment effect at a two-sided 0.05 significance level. In order to account for the potential loss to follow up and the possibility that the effect size may differ based on using sucrose rather than maltodextrin as the carbohydrate challenge, we aimed to recruit 40 participants to complete at least 36 participants.

### Experimental protocol

The protocol used was an adaptation from that described by Brouns et al.[19] and was carried out in accordance with ISO standards [20]. On the day prior to a test, participants were asked to restrict their intake of alcohol and caffeine-containing drinks and to restrict their participation in intense physical activity (for example, long periods at the gym, excessive swimming, running, aerobics). Participants were also told not to eat or drink after 9.00 pm the night before a test, although water was allowed in moderation.

At the start of the test period, participants consumed the products at a comfortable pace, within 5 min and the reference product and test products were served dissolved in 250 mL water. Participants remained sedentary during each test session and did not consume any additional food or fluid.

Blood samples were taken at − 5 min and 0 min before consumption of the treatments and the baseline value taken as a mean of these two values. The products were consumed immediately after this and further blood samples were taken at 15, 30, 45, 60, 90 and 120 min after starting to drink the test/reference product. Blood was obtained by finger-prick using the Unistik^®^3 single-use lancing device (Owen Mumford). Fingers were not squeezed to extract blood from the fingertip as this may dilute with plasma. Blood glucose was measured using the HemoCue Glucose 201 + analyser (HemoCue^®^ Ltd), which is a photometric enzyme coupled assay system. The glucose analyser was calibrated daily using control solution from the manufacturer. Plasma insulin was measured from 300 μL of capillary blood (from finger pricks). Blood was collected into chilled microvette^®^ capillary blood collection tubes treated with dipotassium EDTA (CB 300 K2E; Sarstedt Ltd). The microvette^®^ tubes were centrifuged and 200 μL of the supernatant plasma obtained. Insulin concentrations in the plasma samples were determined by electrochemiluminescence immunoassay using an automated analyzer (Cobas^®^ E411; Roche diagnostics).

### Statistical analysis

Data were analysed using IBM Statistical Package for the Social Sciences (SPSS) version 22.0 (SPSS Inc., Chicago, Illinois). Data are presented as mean, standard deviation (SD) and standard error of the mean (SEM) values. The blood glucose and plasma insulin iAUC for both Reducose^®^ and the placebo were calculated geometrically by applying the trapezoid rule, according to the ISO standards[20]. The peak glucose, peak insulin, time of peak glucose and time of peak insulin were calculated as the average of the peak values for the group of participants after consuming the placebo and test products. Prior to statistical analysis, the normality of the data was tested using the Shapiro-Wilks statistic. The paired-sample t-test (for normally distributed data) and non-parametric Wilcoxon signed-rank test (where data were not normally distributed) were used to compare blood glucose and insulin concentrations, peak blood glucose and insulin values, time of the blood glucose and insulin peaks and blood glucose and insulin iAUC (over 60, 90 and 120 min) between Reducose^®^ and the placebo. Statistical significance was set at p < 0.05.

## Results

Thirty-eight healthy participants were recruited (16 male, 22 female; aged 20 to 58 years). Thirty-seven participants completed the study. One participant was unable to complete the study within the allocated timeframe and was withdrawn from the study. For one participant, data were missing for plasma insulin and therefore excluded from the analysis. Thus, the glycaemic and insulinaemic response data are reported for 36 participants. The physical characteristics of the study population with complete GR and IR data are presented in Table 1.

### Glycaemic and insulaemic response

**Fig. 1:**
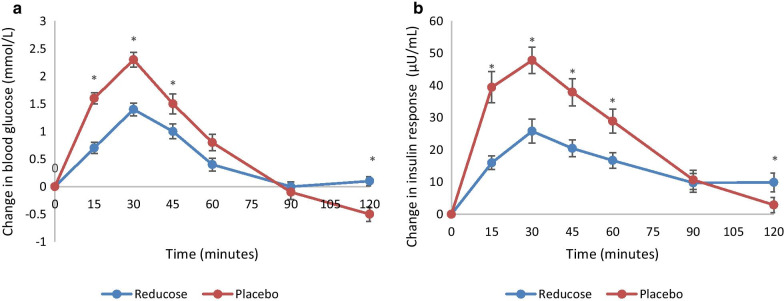
**a **Glucose iAUC for Reducose^®^ and the placebo. **b** Insulin iAUC for Reducose^®^ and the placebo. Values are the mean for 36 participants with SEM asterisks show statistical significance (*P* < 0.05)

There was a significant difference (*p* < 0.05) in the change in blood glucose measurements between Reducose^®^ and the placebo at 15, 30, 45 and 120 min; however, there was no significant difference (*p* > 0.05) in the change in blood glucose measurements between Reducose^®^ and the placebo at baseline, 60 and 90 min (Fig. 1a). After consuming the placebo, the blood glucose at 120 min dropped below the baseline whereas Reducose sustained the blood glucose just above the baseline at the end of two hours. Reducose^®^ significantly lowered the peak blood glucose compared with the placebo, however the time of the blood glucose peak was not significant (Table 2). There was a significant difference between the mean glucose iAUC at 60, 90 and 120 min between Reducose and the placebo ranging from 42 to 45% (Table 3).

**Table 2:** Peak blood glucose, time of the blood glucose peak, peak insulin and time of the insulin peak (*n* = 36)

	Placebo	Reducose^®^	*p *value	Change compared to Placebo (%)
Peak blood glucose (mmol/l)	7.2 ± 0.7	6.2 ± 0.7	< 0.001^a^	− 40^b^
Time of blood glucose peak (min)	27.5 ± 9.1	31.3 ± 7.5	0.050	+ 12
Peak insulin (μU/ml)	62.77 ± 29.87	37.27 ± 22.58	< 0.001^a^	− 41^b^
Time of the insulin peak (min)	31.7 ± 14.7	40.8 ± 22.6	0.149	+ 22

^a^Statistically significant difference (P < 0.05). Values are Mean (± SD)

^b^Based on incremental changes that have been adjusted to baseline values

**Table 3:** Mean (± SD) glucose iAUC and insulin iAUC at 60, 90 and 120 min (n 36)

	Placebo	Reducose^®^	*p *value	Change compared to Placebo (%)
Glucose iAUC 60	88.6 ± 35.4	48.9 ± 23.0	< 0.001^a^	− 45
Glucose iAUC 90	104.3 ± 51.4	58.7 ± 32.7	< 0.001^a^	− 44
Glucose iAUC 120	110.2 ± 63.9	64.0 ± 37.4	0.001^a^	− 42
Insulin iAUC 60	2094.64 ± 1003.13	1060.07 ± 618.91	< 0.001^a^	− 49
Insulin iAUC 90	2691.44 ± 1372.52	1457.22 ± 822.51	< 0.001^a^	− 46
Insulin iAUC 120	2915.94 ± 1642.54	1752.24 ± 1013.20	< 0.001^a^	− 40

^a^Statistically significant difference (*p* < 0.05). Values are Mean (± SD)

There was a significant difference (*p* < 0.05) in the change in insulin measurements between Reducose^®^ and the placebo at 15, 30, 45, 60 and 120 min; however, there was no significant difference (*p* > 0.05) in the change in insulin measurements between Reducose^®^ and the placebo at baseline and at 90 min (Fig. 1b). The insulin measurement dropped towards the baseline at 120 min after the placebo. After Reducose, the insulin measurement was maintained above the baseline at 120 min. Reducose significantly lowered the peak plasma insulin levels compared with placebo, however the time to the peak was not significantly different (Table 2). There was a significant difference (*p* < 0.05) in the plasma insulin iAUC between Reducose^®^ and the placebo at 60, 90 and 120 min ranging from 40 to 49% (Table 3).

The results demonstrated that blending Reducose directly into the test meal positively impact efficacy with the reduction in peak postprandial glucose being 42% lower (p < 0.001) and postprandial plasma insulin being 41% lower (p < 0.001) than the placebo arm. There were no serious adverse events or adverse events during this study.

## Discussion

Previous research [17, 18] has demonstrated that white mulberry extracts can lower postprandial glucose and insulin levels following ingestion of long-chain carbohydrates, such as maltodextrin. Carbohydrates such as maltodextrin are glucose polymers, with each glucose unit joined to the next one in the chain through an α-1,4-glycosidic bond. These carbohydrates are digested by enzymes such as amylase and α-glucosidase. Sucrose however is a disaccharide made up of one molecule of glucose that is bonded through an α-1,2-glycosidic bond to one molecule of fructose. Due to the different bond orientation, sucrose is not digested by amylase or α-glucosidase but is rather broken down to its basic monosaccharides through the actions of the enzyme sucrase or sucrase-isomaltase [21].

In this randomised, double blind, placebo-controlled clinical trial, Reducose^®^ mulberry leaf extract significantly lowered postprandial increases in blood glucose and plasma insulin in normoglycaemic individuals following the ingestion of sucrose. The results showed a clear and significant effect of white mulberry leaf extract to decrease both glucose and insulin concentrations following a sucrose challenge. The study showed that both the peak blood glucose and blood insulin levels were lowered, as was the total postprandial glucose and insulin levels, as represented by iAUC.

The mulberry leaf extract group exhibited a more balanced glucose response, with the participant’s blood glucose levels never dropping below the fasted level, whilst the placebo group showed a significantly lower blood glucose level at the 120-min time point (p < 0.001), levels lower than the fasted baseline. This transient reactive hypoglycaemia in the placebo group may be attributed to the rapid increase in blood glucose and the consequent spike in plasma insulin. In non-diabetic subjects, incretins are responsible for 50–70% of total insulin secretion. Glucose-dependent insulinotropic peptide (GIP) and glucogon-like peptide-1 (GLP-1) both play an important role in glucose homeostasis[22], but the rate of release of these incretins differs depending on the stimulatory nutrient. GIP is synthesized by K-cells in the duodenum and jejenum and is very sensitive to glucose stimulation but has lower sensitivity to disaccharides and polysaccharides. GLP-1, which is produced by the L-cells that predominate at the distal end of the small intestine in the ileum, shows similar stimulatory sensitivity to glucose as to disaccharides and polysaccharides[23]. In healthy subjects, GIP is responsible for the majority of postprandial incretin-mediated insulin response[22] and in situations with lower amounts of free glucose, there would be a corresponding lower GIP-mediated insulin response. This may explain the differences in blood glucose responses between the test groups in the current study and is in line with the established inhibitory effect that mulberry leaf extract has on glucosidase enzymes in the brush border[24]. In the placebo group, the sucrose test drink would be rapidly hydrolysed to glucose and fructose by glycosidase enzymes in the brush border of the intestine, resulting in a higher insulin release due to stimulation by both GIP and GLP-1, whilst in the Reducose^®^ group, lower levels of free glucose would be available due to the inhibition of the hydrolysing enzymes, resulting in lower GIP levels. Early postprandial reactive hypoglycaemia, which occurs within 1–2 h of eating, can be caused by an exaggerated incretin effect as excessive amounts of GIP and GLP-1 are released through the sudden glucose loading, stimulating insulin exocytosis and upregulation of GLUT-4 channels, which results in a rapid lowering of blood glucose levels[25].

This study also found that the mulberry leaf extract group also had significantly higher insulin levels at 120 min (p < 0.001). This finding may again be attributable to an incretin effect and reflect the malsorption of the sucrose test meal. The majority of carbohydrate digestion takes place in the duodenum and early parts of the jejenum, where there is a higher concentration of glucosidase enzymes in the brush border, with the more distally situated jejenum and ileum being sites of nutrient absorption[25]. There is consequently limited scope for digestion and absorption of disaccharides and polysaccharides that have escaped break-down in the proximal small intestine, as only monosaccharides can be absorbed into the body. These undigested nutrients may however stimulate the L-cells in the ileum resulting in secretion of GLP-1. GLP-1 has number of physiological actions, one of which is glucose stimulated insulin release, and the rise in insulin levels that are observed in the mulberry leaf extract treatment arm after 90-min may be attributed to the stimulation of GLP-1 due to sucrose malsorption. This supposition is supported by literature as reported small bowel transit times correlate with the rise in insulin after 90 min[26] and treatment with α-glucosidase inhibitors such as acarbose are associated with increased postprandial GLP-1 levels[27].

White mulberry leaf extract has broad applications in blunting the blood glucose responses of foods that contain a range of dietary carbohydrates, with the results from this study showing that mulberry leaf extract is effective in lowering postprandial glucose rises after consuming α-1,2 bonded carbohydrates in addition to being effective in response to α-1,4 bonded carbohydrates as demonstrated previously[18]. In comparison other functional ingredients that impact the digestion of carbohydrates, such as white kidney bean extract (reported efficacious dose ranges from 750 mg-3000 mg), only inhibit α-amylase enzymes (hydrolyse α-1,4 bonded polysaccharides into oligo- and disaccharides) and therefore would not prevent the breakdown of sucrose into its carbohydrate monomers[28].

The findings of this study agree with previous research that has demonstrated the sucrase inhibitory effect of white mulberry leaf extracts in healthy and type-2 diabetic participants[29, 30]. However, the ratio of mulberry leaf extract to sucrose was higher (3 g mulberry leaf extract with 30 g sucrose; 1 g mulberry leaf extract with 75 g sucrose) in those studies[29, 30] compared with the current study where the dose of Reducose^®^ mulberry leaf extract was lower due to greater levels of purification (250 mg mulberry leaf extract with75 g sucrose). Similar to Mudra et al. [29], the use of pure sucrose as a test food in this study averted any interfering effects from other ingredients in the Japanese confections used by Nakamura et al. [30]. Furthermore, another strength of this study is the larger sample size compared to previous studies investigating the effect mulberry leaf extracts on sucrose digestion and absorption [29, 30].

Previous research by this research group [18] reported that the choice of dosage format could impact the efficacy of mulberry leaf extracts. In the previous study, the test product did not have a notable effect in lowering glucose or insulin responses until 45 min after ingestion and consequently the peak glucose and insulin levels were not decreased to the anticipated level as peak levels occurred at 30 min. It was hypothesized that the capsule material selected for use in the study (hydroxypropyl methylcellulose), which in this case had a 15-min disintegration time, impaired the bioavailability of the mulberry leaf extract as blood glucose in the test participants had already started to peak before mulberry leaf extract was bioavailable to inhibit the glycosidase enzymes. To explore this hypothesis further, in the current study, the mulberry leaf extract was blended directly into the carbohydrate challenge and dissolved in water so that it would be immediately bioavailable. The results demonstrated that blending mulberry leaf extract directly into the test meal positively impacts efficacy with the reduction in peak postprandial glucose being 42% lower and postprandial plasma insulin being 41% lower than the placebo arm, compared with a non-significant, 3% reduction reported in Lown et al. [18]. This suggests that in order to get the best effect from mulberry leaf extract, a suitable intake form such as liquids, fast-dissolving tablets or capsules, or chewable dosage forms should be considered during the product development when mulberry leaf extract is used as a key ingredient.

The findings of the current study show that mulberry leaf extract could be beneficial for people who are trying to control their blood glucose response to foods. As noted previously, the DPP study reported that lifestyle changes had the greatest impact in preventing diabetes in participants that had IGT [8, 9] and preventing the development of latent or overt T2DM is of growing importance considering the increased risk factors associated with COVID-19 [3]. Changing health-related behaviour is however difficult as there are both social and psychological factors that need to be modified before such changes can be made [31]. Eating and physical activity are processes and practices that are embedded in social life and individual habits in eating and exercising are ingrained in an individual’s routine. Whilst most people are rational, that does not always equate to rational behaviour. Most smokers want to stop smoking and most overweight people want to lose weight, but the majority of smokers do not stop and most diets fail because knowledge and rational assessment does not drive behavioural change, as behaviour is largely automatic and usually unaccompanied by conscious reflection [31, 32]. Small, incremental changes or nudges are more likely to result in health behaviour changes [32, 33]. For people who are concerned about their blood glucose levels, using dietary supplements such as mulberry leaf extract to blunt the glycaemic and insulinaemic responses to food could be considered such a small incremental change or nudge.

## Limitations

This study only evaluated the short-term effects of mulberry leaf extract using a single dose and therefore did not capture any benefits or potential side effects that could occur with long-term administration. We also used a simple carbohydrate in fasting individuals and did not evaluate the pragmatic effects of mulberry leaf extract when carbohydrates are mixed with fats and proteins in the diet as proteins and fats may impact carbohydrate digestion and the rate of gastric emptying. Whilst a study in a complex meal helps to establish real-world benefits (this is currently being explored in an ongoing clinical study), understanding the effects of Reducose^®^ on the different types of carbohydrates is nonetheless important due to the variety of carbohydrates in the human diet, each of which contributes to the glycaemic response but require different enzymes for digestion. As this study was conducted in normoglycaemic participants, care should be taken when extrapolating the results to participants with dysglycaemia.

## Conclusions

We have demonstrated that Reducose^®^ mulberry leaf extract significantly lowers the increase in plasma glucose after ingestion of sucrose over 120 min. Total insulin rises were also significantly suppressed over the same period. None of the participants reported any side effects and no adverse events were recorded during the study. This study builds on the body of evidence that supports the use of mulberry lead extracts as a part of lifestyle changes that may lead to healthy blood glucose levels.

## Data Availability

The datasets generated and analysed in the current study will be available to researchers upon request from Phynova Group Limited. Anonymised patient level data will be available for research purposes within six months of publishing the results of the primary endpoints, key secondary endpoints and safety data in a peer-reviewed journal. As participants only consented for their data to be used to investigate a particular health benefit of the test product, access to data will only be provided for researchers that are investigating the same or an equivalent product for the same health benefit. Data will be made available on request provided Phynova has the legal authority to provide the data; for example, if Phynova has out-licensed the data to another company then it would no longer have the legal authority to provide the data. Data will only be made available if Phynova is able to anonymise the data without compromising the confidentiality and privacy of research participants. The following information will be made available: raw dataset excluding any patient images, protocol with any amendments, analysis and reporting plan. Researchers requesting access to data should email their requests to info@phynova.com and should include a description of the intended research.

## Abbreviations

BMI: Body mass index
DNJ: 1-Deoxynojirimycin
DPP: Diabetes Prevention Program
GR: Glycaemic response
iAUC: Incremental area under curve
IFG: Impaired fasting glucose
IGT: Impaired glucose tolerance
IR: Insulinaemic response
NCD: Non-communicable disease
PPG: Post-prandial glucose
SEM: Standard error of mean
SD: Standard deviation
T2DM: Type-2 diabetes mellitus
UREC: University research ethics committee
