# Correction to: Mulberry leaf extract improves glycaemic response and insulaemic response to sucrose in healthy subjects: results of a randomized, double blind, placebo‑controlled study

**DOI:** 10.1186/s12986-021-00577-w

**Published:** 2021-05-26

**Authors:** Pariyarath Sangeetha Thondre, Helen Lightowler, Lis Ahlstrom, Andrew Gallagher

**Affiliations:** 1 Oxford Brookes Centre for Nutrition and Health, Faculty of Health and Life Sciences, Oxford Brookes University, Gipsy Lane Campus, Headington, Oxford, OX3 0BP UK; 2 Phynova Group Ltd, 16 Fenlock Court, Blenheim Office Park, Long Hanborough, OX29 8LN UK

## Correction to: Nutr Metab (Lond) (2021) 18:41 https://doi.org/10.1186/s12986-021-00571-2

Following the publication of the original article [1], errors were identified in the reference.

The changes have been highlighted in **bold typeface**.

**Reference**

[20] Food Products-Determination of the glycaemic index (GI) and recommendation for food classification. ISO **26642:** 2010**(E)**.
